# Hepatitis B Virus Genotypes in the Kingdom of Bahrain: Prevalence, Gender Distribution and Impact on Hepatic Biomarkers

**DOI:** 10.3390/medicina55100622

**Published:** 2019-09-23

**Authors:** Essam M. Janahi, Zahra Ilyas, Sara Al-Othman, Abdulla Darwish, Sanad J. Sanad, Budoor Almusaifer, Mariam Al-Mannai, Jamal Golbahar, Simone Perna

**Affiliations:** 1 Department of Biology, College of Science, University of Bahrain, Shakir P.O. Box 32038, Bahrain; zahra.muhammadilyas@gmail.com; 2 Molecular Diagnostic Al-Jawhara Centre for Molecular Medicine and Inherited Disorders, AGU, Manama 329, Bahrain; sarash@agu.edu.bh; 3 Department of Pathology, Bahrain Defense Force Hospital, West Riffa P.O. Box 28743, Bahrain; abdulla.darwish660@gmail.com (A.D.); b.mm89@hotmail.com (B.A.); 4 Department of Internal Medicine, Bahrain Defense Hospital, West Riffa P.O. Box 28743, Bahrain; Sanad.jassim@outlook.com; 5 Department of Mathematics, College of Science, University of Bahrain, Shakir P.O. Box 32038, Bahrain; malmannai@uob.edu.bh; 6 Department of Clinical Biochemistry, Northern Devon NHS Trust, North Devon District Hospital, Barnstaple EX31 4JB, UK; jgolbahar@nhs.net

**Keywords:** prevalence, hepatitis B virus, genotype, bahrain

## Abstract

*Background*: Approximately 400 million people are infected with Hepatitis B virus (HBV) around the world, which makes it one of the world’s major infectious diseases. The prevalence of HBV genotypes and predictive factors for risk are poorly known in the Kingdom of Bahrain. *Objectives*: The aim of the present study was to investigate the prevalence of HBV genotypes, its correlation with demographic factor sand impacts on hepatic biomarkers. *Materials and Methods*: Venous blood samples were collected from 82 HBV positive patients (48 males, 34 females). The extraction of HBV DNA, PCR amplification, and genotyping were done to classify different genotypes (A, A/D, B, B/D, C, D, D/E, E). HBV genotypes association with gender, nationality, mode of transmission, and liver cirrhosis complication was determined by descriptive statistic and univariate analysis of variance (ANOVA). For liver function test, unpaired t-test and ANOVA were performed. *Results:* The predominant genotype among patients under study was genotype D (61%), followed by genotype A (10%), and lowest frequency was found for undetermined genotype (1%). In general, there was no significant association between the different genotypes and some demographical factors, serological investigations, and liver function test. The prevalence of HBV genotypes was higher in male patients as compared to female patients and higher in non-Bahraini than in Bahraini. Patients with the dominant genotype D showed higher than the normal maximum range for alanine aminotransferase (ALT) (mean = 45.89) and Gamma-glutamyl transferase (GGT) (mean = 63.36). *Conclusions*: The most common HBV genotype in Bahrain was genotype D, followed by genotype A. Further studies involving the sources of transmission and impact of hepatic biomarker in Bahrain are required to enhance the control measures of HBV infections.

## 1. Introduction

Hepatitis B Virus (HBV) is chronically carried by around 400 million people worldwide and about one million die annually as result of developing liver cirrhosis and hepatocellular carcinoma [1,2,3]. The infection is mainly present in Middle East, South-East Asia, sub-Saharan Africa, Central and South-America, and Eastern-Europe with prevalence >8% of population [4]. A migratory flow that had occurred in last twenty years from these countries to the industrialized countries resulted in an increase in HBV prevalence the industrialized countries [1,5]. Between 5 and 10% of infected individuals become chronic carriers in their adulthood, while 85 to 95% in their infancy [6].

Hepatitis B virus is transmitted through blood and body fluids, hence certain types of behaviors increase the risk of infection, such as sharing personal items (toothbrushes, razors, etc.), use of contaminant needles for intravenous drugs or ear pricing and tattooing, and practicing unsafe sex. Hemodialysis and hemophiliacs patients as well as Healthcare and emergency service workers are also at higher risk [7].

HBV genome has a high rate of mutation when compared to other DNA viruses due to the high spontaneous error rate of the viral reverse transcriptase and lack of proofreading mechanism. It is estimated approximately 1.4–3.2 × 10^−5^ per genome. Accordingly, HBV can be classified into eight genotypes A-H that accounts for 8% or more in the complete nucleotide sequence on inter-sequence divergence [8,9]. Studies on HBV genotypes show a distinct geographical distribution around the world [10]. In general, genotype A is pandemic, but most prevalent in North West Europe, North America, Central Africa [11], and India [8,12]. Genotypes B and C are prevalent in Asia [1,13], especially in the populations of Eastern Asia and the Far East [3]. Genotype D is distributed worldwide with the highest prevalence in the Mediterranean region [3,9,14]. Genotype E and F are predominant in West Africa and in the Amerindian population, respectively [1,9,13]. Recently, genotype G was identified in the USA and France [1]. Genotype H was also recently found in Central America [15]. A remarkable difference in the clinical and virologic characteristics between the patients with different genotypes has been reported [2].

HBV genotypes are reported to be responsible for the differences in the natural history of chronic infection and they play a significant role in clinical manifestation of infection and response to antiviral therapy [16]. Therapeutically, patients that are infected with genotypes A, B, D, and F show frequent spontaneous HBeAg seroconversion when compared to genotype C. Whereas, patients that were infected with genotype E have higher frequency of HBeAg positivity and higher viral loads as compared to patients that were infected with genotype D [17].

Epidemiological data regarding HBV in any country would provide significant information to program managers and health planers to control and manage the infection with reference to its etiological spectrum. In the present study, we aim to determine the prevalence of the various HBV genotypes in the Kingdom of Bahrain. This study also aims to determine various sociodemographic factors and hepatic biomarker associated with the prevalence and the possible risk factors for HBV transmission in Bahrain.

## 2. Method

### 2.1. Setting

Bahrain is a small archipelago country (33 islands) that is situated near the western shores of the Arabian Gulf with a total area of 765.3 km^2^. It has a total population of 1,234,571, out of which 666,172 are non-Bahraini and it is one of the most densely populated countries in the world (1461/km^2^) [18].

### 2.2. Study Design

In this cross-sectional study, patients with established chronic hepatitis B infection (positive for HBsAg antigen, HBcAg antibody) referred to Bahrain Defense Force Hospital (second largest hospital in Bahrain) were investigated for HBV genotypes. The major inclusion criterion was testing positive for HBsAg for over six months (chronic infection), with levels of ALT around the normal range. All patients were negative for antibodies against hepatitis C and human immunodeficiency virus.

### 2.3. Sample Collection, HBV DNA Extraction and Genotyping

Five ml of venous blood were collected from patients and sera were separated and stored at −80 °C prior to HBV genotyping. HBV DNA extraction, PCR amplification of DNA, and genotyping were carried out according to the instruction while using a commercially available kit (SMITEST, MBL Co. Nogoya, Japan) based on hybridization with type-specific probes immobilized on a solid-phase support. The quality and quantity of extracted DNA was determined with a Nano drop spectrophotometer (Thermo Scientific, Wilmington, DE, USA) after DNA extraction. Serological investigation for HBsAg and HBeAg were carried out while using commercial enzyme-linked immunosorbent assay (ELISA) kits (ARCHITECT ANALYSER i2000, Abbott, Santa Clara, CA, USA). Liver function tests; Total and Direct Bilirubin, Alanine Transaminase (ALT), Alkaline Phosphatase (ALP), Aspartate Transaminase (AST), Gama Glutamyl Transferase (γGT) and Lactate Dehydrogenase (LDH) were analyzed in serum samples while using routine biochemistry analyzer (COBAS c 501, Roche/ Mannheim, Germany).

### 2.4. Statistical Analysis

Statistical analyses were performed while using IBM SPSS Statistics 23 software. For baseline variables, summary statistics employed frequencies data, mean, and standard deviation (SD) for continuous variables. Continuous variables were compared using unpaired t-tests and ANOVA as appropriate. Descriptive Statistics were calculated to determine the prevalence of genotype with respect to data of demographic factors, serology, and other investigations. Univariate analysis of Variance (ANOVA) was applied to determine any significance differences for the genotype and liver function tests.

### 2.5. Ethical Approval

The local ethics and research committees of Bahrain Defense Force Hospital and University of Bahrain approved this study (approval date 7 September 2017, Project code: 2017/12). It conformed to the provisions of the Declaration of Helsinki in 1964 (and revised in Fortaleza, Brazil, October 2013). All of the patients signed the informed consent form before participation.

## 3. Results

### 3.1. Demographic Characteristics of Patients

Eighty-two HBV positive patients (48 males, 34 females), were screened for the different genotypes A, B, C, D, E and mixed genotype infections A/D, B/D, D/E. The patients in the study were either Bahraini nationals or from other nationalities. The frequency of Bahraini was found to be 53.7% and that of Non-Bahraini was 46.3%. The highest prevalence of HBV infection was shown for the age group 21–30. The lowest prevalence was for the group <21. There was no significance difference between genotype and nationality, gender and age-group (*p* > 0.05), as shown in Table 1.

### 3.2. Distribution of HBV Genotypes in the Study Population

**Figure 1:**
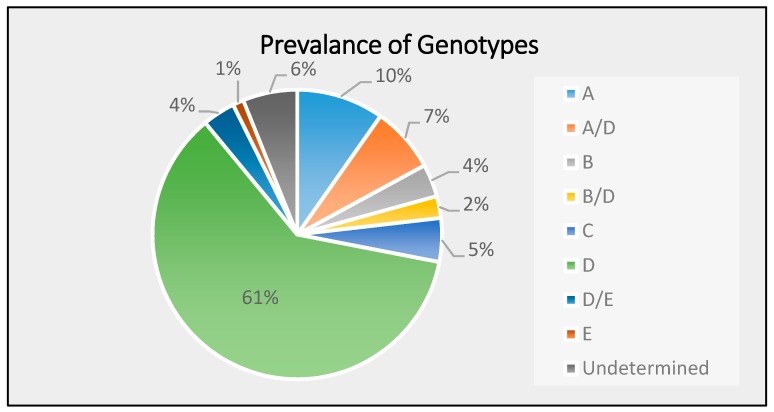
Prevalence of HBV genotypes among 82 patients under study in the Kingdom of Bahrain.

Figure 1 illustrates the frequency of HBV genotypes A, A/D, B, B/D, C, D, D/E, and E. The prevalence of genotype A was found to be 10%, genotype A/B was 7%, genotype B was 4%, genotype B/D was 2%, genotype C was 5%, genotype D/E was 4%, genotype E was 1%, and undetermined genotypes was 6%. Among the referred genotypes, genotype D showed the highest occurrence (61%), which indicated that genotype D is the most prevalent HBV genotype in the Kingdom of Bahrain.

### 3.3. Genotype and Sociodemographic Data

Table 2 states the distribution of genotypes with respect to gender, nationality, and age groups. The prevalence of HBV infection for each genotype differs for gender, but there is no significance difference in terms of genotype prevalence and genders (*p* = 0.447; *p* > 0.05). Overall, the genotype A (62.5%), A/D (66.7%), B (66.7%), C (100%), and D/E (100%) are more related to males, whereas genotype E (100%) and Undetermined genotype (60%) are more related to females. Genotype A/D (66.7%), D (64.0%), and Undetermined genotypes (80%) are more related to Bahraini nationality; Genotype A (87.5%), B (6.7%), C (75%), D/E (100%), and E (100%) are more related to other nationalities. Genotype B/D (50%) is equally distributed among and Bahrainis and non-Bahrainis. Individuals of different age groups were enrolled in the study, there was no significance differences in terms of genotype prevalence and age (*p* = 0.409; *p* > 0.05).

### 3.4. HBV Genotype Relationship with Mode of Transmission and Liver Cirrhosis Complications

Table 3 shows the prevalence of HBV genotypes in relationship to the mode of transmission and liver cirrhosis complications. There are no significant differences (*p* > 0.05) in genotype frequency in relation to the mode of transmission (*p* = 0.086) and liver cirrhosis complications (*p* = 0.857). Genotype A (37.5%), A/D (66.7%), D (66%), D/E (100%), and undetermined genotype (80%) are more related to the unknown factor of infection transmission. Genotype B (66.7%) and C (75.5%) are more related to Blood transfusion while genotype E (100%) is more related to vertical transmission from mother to child. Genotype D (2%) is more related to hemodialysis, while genotype A (12.5%) is more related to tattoo/body piercing. In general, there was low or no relationship between the prevalence of HBV genotypes and liver cirrhosis complications, for example, genotype A (50%), genotype A/D (66.7%), genotype B (100%), genotype C (100%), genotype D (78%), genotype D/E (100%), and undetermined (80%), except for mixed genotypes B/D, 50% of the patients were associated with ascites.

### 3.5. HBV Genotype and Liver Function Test

For the determination of HBV clinical course, investigating the hepatic biomarkers plays an important role. As increase or decrease in their levels can indicate hepatic disfunction. Bilirubin, Direct Bilirubin (Dbilirubin), Alanine transaminase (ALT), Aspartate aminotransferase (AST), and Gamma-Glutamyl Transferase (GGT) were measured and associated with the different genotypes. Table 4 summaries the mean and SD values in relation to the different genotypes. The maximum and minimum range for these liver function enzymes were stated as: Bilirubin (max. 17, min. 0 umol/L), Dbilirubin (max. 3.4, min. 0 umol/L), AST (max. 37, min. 0 lU/L), ALT (max. 41, min. 0 lU/L), and GGT (max. 49, min. 11 lU/L). Table 4 shows that for Bilirubin value A/D mixed genotype was higher than the max range; for D bilirubin, no genotypes were above or below the normal range, while for AST, ALT, and GGT, the genotypes A, A/D, B, and B/D were all above the normal range.

## 4. Discussion

HBV infection is an important global problem that places a continuously increasing burden on developing countries. As the HBV genotype can be classified into different genotypes, the classification has to be cost-effective and clinically relevant [19]. Research on the relationship between HBV genotypes, their pathogenicity in chronic liver disease, including hepatocellular carcinoma, and their therapy are of great interest, as this allows for understanding the spread and risk of HBV infection around the world [10]. On the other hand, HBV infection is a major health problem in the Middle East. The majority of the countries in the region have an intermediate or high endemicity of HBV infection [20]. Despite the low prevalence of HBV in Bahrain, it is important to investigate the frequency of HBV genotypes and its association with various sociodemographic factors, hepatic biomarkers, and mode of transmission, which is essential for fine tuning the control of the disease.

According to a study by Janahi (2014), completed on 877,892 individuals, Bahrain has low HBV endemicity for the period (2000–2010). The prevalence of hepatitis B virus infection in Bahrain was found to be 0.58% [21]. This study reports for the first time in Bahrain, the correlation of HBV genotypes frequency with the demographic characteristics and hepatic biomarker. The results showed that there were no significant differences of genotype frequency in relation to the demographic characteristics as well as hepatic biomarkers. Out of the 82 screened patients in this study, 58.5% were male, while the remaining 41.5% were females. There was a significant increased risk of HBV infection in male as compared to females (Table 1). 53.7% of HBV positive patients had Bahraini nationality, while the remaining 46.3% belonged to other eleven nationalities, such as Pakistan, Sudan, Egypt, Yamen, Syria, Kuwait, Bangladesh, India, Ethiopia, Indonesia, and Philippines, which are known to be highly endemic for HBV. Relationship between genotype and age-group indicates that HBV prevails 4.9% in <21 years, 28% in 21–30 years, 25.6% in 31–40 years, 13.4% in 41–50 years, 15.9% in 51–60 years, and 12.2% in >61 years. age groups.

The frequency of mode of transmission was highly unknown (59.8%), followed by blood/blood products (19.5%), sexual contact (7.3%), vertical transmission (7.3%), and finally organ transplant (3.7%). HBV and HCV have common modes of transmission; therefore, their coinfection is quite frequent. This particularly occurs in areas where the two viruses are endemic and among subjects with high risk of parental infection [22]. According to a study that was conducted in Bahrain, dental procedures and surgical operations account for 37.2% and 35.6%, respectively, of the HBV transmission routes. Followed by the blood transfusion (24.6%), the sexual contact and intervenors drug abuse were the least possible routes of transmission [21]. There was some significant difference in the HBV genotype prevalence with respect to some investigated variables. For example, the frequency of HBV genotype is more related to males and the risk of HBV infection increased with older age.

The dominant genotype in our study was genotype D with 61% frequency, which is similar to some countries in the Middle East, like Saudi Arabia (81%) [23], UAE (79.5%) [24], Iran, and Jordan (≈100%) [25,26]. The dominance of this genotype might be attributed to different factors, such as the presence of high number of workers from countries that are known to have dominant D genotype, such as India, Pakistan, Yemen, Syria, and Bangladesh. These infected workers are a principal source for the transmission of hepatitis B. As most of them belong to highly endemic countries with low educational and socio-economical backgrounds, they positively contribute to the transmission of the disease. Living in small houses and having unhygienic behaviors (such as sharing same razors and toothbrushes) put such workers at high risk of contracting HBV. A poor hygiene system in hospitals of such countries is known as a high-risk factor for HBV transmission, as the same syringe is used for vaccination of different people [27].

The quasi-species nature of HBV infection indicates that the variation and evolution of Hepatitis B virus has been influenced by the recombination between genotypes. Hence, a high prevalence of more than one dominant genotype in a certain region is common [16,28]. It is documented that mixed infection with different HBV genotypes is not uncommon and it is of great virological and clinical interest. For example, a study done by Chen et al. (2004) showed that the prevalence of mixed HBV genotype infection was 16.3% for HBsAg positive and 34.4% in occult HBV-infected intravenous drug users [29].

HBV is non-cytopathic virus, which highlights the complex and important interaction between the virus and host in causing HBV-related liver disease. Bilirubin, Direct Bilirubin, ALT, and AST are the most common liver enzymes that are measured to investigate the condition of liver due to HBV or HCV infection [30]. Elevated ALT levels, elevated AST level, elevated serum bilirubin, and decreased serum albumin might be indicative of advanced liver disease and even cirrhosis [31]. In our study, there was no significant association between HBV prevalence and liver function test. However, each genotype showed variation depending on the mean and standard deviation of the liver function test associated with that genotype. The most dominant genotype in the present study, genotype D showed high levels of ALT and GGT above the normal range. This might be indicative of acute hepatitis. Genotype A and mixed genotypes B/D showed higher than the normal maximum level for ALT, AST, and GGT; mixed genotypes A/D showed higher than the normal maximum level for Bilirubin, ALT, AST, and GGT; genotype B showed higher than the normal maximum level for ALT and GGT; genotype D showed higher than the normal maximum level for ALT and GGT; and, genotype E showed higher than the normal maximum level for AST and ALT. Genotype C and undetermined genotypes showed normal liver function enzymes levels. It is also reported that persistently elevated liver enzymes levels in an asymptomatic hepatitis B patient is associated with high infectivity [32].

This study, as any study, has some limitations. Firstly, 82 individuals only were screened, which is a relatively small sample and it may not represent an accurate picture of HBV prevalence at the population level. Secondly, no cases from other governmental and private health facilities were included in our study which may contribute to lower prevalence. However, according to the latest data that were obtained from the Public Health Directorate, Ministry of Health, the number of HBV positive patients at population level was 527, so our sample 82 represents approximately 16% of the total HBV patients in Bahrain. Thirdly, some of the obtained data from patients were based on patient self-reporting of risk factors, which is subject to social desirability bias.

Finally, this study showed that the overall HBV prevalence among males’ patients to be 58.5%, while it was 41.5% among females. The dominant HBV genotype in Bahrain was genotype D (61%), which was associated with higher than the normal maximum level of ALT and GGT.

## 5. Conclusions

This study highlighted the importance of hepatic biomarker association with genotypes, which can be used as a base for further studies to investigate such an association. The advantage of this study was to provide a baseline study to draw a good estimate of HBV genotype distribution in Bahrain.

## Figures and Tables

**Table 1:** Sociodemographic factors associated with the prevalence of hepatitis B virus infections examined patients (N = 82).

Variables	No. of Isolation ^1^	*p*-Value
Gender		0.447
Male	58.5 (48)	
Female	41.5 (34)	
Nationality		0.44
Bahraini	53.7 (44)	
Non-Bahraini	46.3 (38)	
Age-group		0.409
<21	4.9 (4)	
21–30	28 (23)	
31–40	25.6 (21)	
41–50	13.4(11)	
51–60	15.9(13)	
>61	12.2(10)	

^1^ Data presented as %(No.).

**Table 2:** Prevalence of Hepatitis B Virus (HBV) Genotypes in Patients of Different Gender, Age and Nationality (N = 82).

	Gender		Nationality		Age-Group					
Genotype	Male (48)	Female (34)	Bahraini (44)	Non-Bahraini (38)	<20 (4)	21–30 (23)	31–40 (21)	41–50 (11)	51–60 (13)	>61 (10)
A	62.5%	37.5%	12.5%	87.5%	0.0%	25.0%	25.0%	25.0%	12.5%	12.5%
A/D	66.7%	33.3%	66.7%	33.3%	0.0%	83.3%	16.7%	0.0%	0.0%	0.0%
B	66.7%	33.3%	33.3%	66.7%	0.0%	0.0%	33.3%	0.0%	33.3%	33.3%
B/D	50.0%	50.0%	50.0%	50.0%	0.0%	0.0%	100.0%	0.0%	0.0%	0.0%
C	100.0%	0.0%	25.0%	75.0%	0.0%	25.0%	50.0%	25.0%	0.0%	0.0%
D	54.0%	46.0%	64.0%	36.0%	8.0%	22.0%	20.0%	14.0%	22.0%	14.0%
D/E	100.0%	0.0%	0.0%	100.0%	0.0%	100.0%	0.0%	0.0%	0.0%	0.0%
E	0.0%	100.0%	0.0%	100.0%	0.0%	100.0%	0.0%	0.0%	0.0%	0.0%
Undetermined	40.0%	60.0%	80.0%	20.0%	0.0%	0.0%	60.0%	20.0%	0.0%	20.0%

**Table 3:** Frequency of HBV Genotypes in Correlation with Mode of Transmission and Liver Cirrhosis Complications.

		Mode of Transmission						Liver Cirrhosis		
Genotype	Unknown (49)	Blood/Blood Products (16)	Sexual (6)	Maternal/Vertical (6)	Organ Transplant (3)	None (63)	Ascites (13)	Portal HTn (2)	HCC (2)	Hepatic Encephalopathy (2)
A	37.50%	12.50%	25.00%	12.50%	0.00%	50.00%	37.50%	12.50%	0.00%	0.00%
A/D	66.70%	16.70%	0.00%	0.00%	16.70%	66.70%	0.00%	0.00%	16.70%	16.70%
B	0.00%	66.70%	0.00%	0.00%	33.30%	100.00%	0.00%	0.00%	0.00%	0.00%
B/D	50.00%	0.00%	50.00%	0.00%	0.00%	50.00%	50.00%	0.00%	0.00%	0.00%
C	25.00%	75.00%	0.00%	0.00%	0.00%	100.00%	0.00%	0.00%	0.00%	0.00%
D	66.00%	16.00%	6.00%	8.00%	2.00%	78.00%	16.00%	2.00%	2.00%	2.00%
D/E	100.00%	0.00%	0.00%	0.00%	0.00%	100.00%	0.00%	0.00%	0.00%	0.00%
E	0.00%	0.00%	0.00%	100.00%	0.00%	100.00%	0.00%	0.00%	0.00%	0.00%
Undetermined	80.00%	20.00%	0.00%	0.00%	0.00%	80.00%	20.00%	0.00%	0.00%	0.00%

**Table 4:** Mean and Standard Deviation of Liver Function Tests, Univariate Analysis with HBV Genotype.

Genotypes	Bilirubin (umol/L)	Dbilirubin (umol/L)	AST (lU/L)	ALT (lU/L)	GGT (lU/L)
A	14.39 (±3.65)	6.94 (±2.81)	44.04 * (±3.78)	49.54 * (±8.77)	65.75 * (±23.22)
A/D	25.10 * (±8.48)	12.78 (±4.79)	77.43 * (±3.68)	94.23 * (±8.89)	65.33 * (±28.07)
B	16.63 (±12.35)	8.97 (±6.77)	35.40 (±14.61)	63.93 * (±38.93)	60.00 * (±39.68)
B/D	16.35 (±11.05)	10.55 (±8.35)	69.40 * (±0.00)	86.90 * (±69.80)	76.00 * (±67.00)
C	6.68 (±2.18)	1.93 (±0.39)	20.45 (±3.15)	21.53 (±5.97)	41.25 (±15.26)
D	13.26 (±1.51)	6.09 (±0.62)	31.76 (±3.05)	45.89 * (±6.60)	63.36 * (±2.68)
D/E	7.70 (±2.99)	3.30 (±1.14)	24.93 (±3.58)	33.53 (±6.63)	25.33 (± 7.88)
E	3.20 (±0.00)	1.50 (±0.00)	37.00 * (±0.00)	44.50 * (±0.00)	15.00 (±0.00)
Undetermined	7.14 (±1.41)	2.50 (±0.25)	18.80 (±2.69)	21.32 (±4.45)	18.00 (±4.06)

* Refers to the measured levels of the liver functions enzymes above their min. and max. ranges.
